# Cerebrovascular impedance estimation with near-infrared and diffuse correlation spectroscopy

**DOI:** 10.1117/1.NPh.10.1.015002

**Published:** 2023-01-23

**Authors:** Jason Yang, Alexander Ruesch, Jana M. Kainerstorfer

**Affiliations:** a Carnegie Mellon University, Department of Biomedical Engineering, Pittsburgh, Pennsylvania, United States; b Carnegie Mellon University, Neuroscience Institute, Pittsburgh, Pennsylvania, United States

**Keywords:** cerebrovascular impedance, cerebrovascular resistance, diffuse correlation spectroscopy, near infrared spectroscopy, cerebral autoregulation, pulsatile hemodynamics

## Abstract

**Significance:**

Cerebrovascular impedance (CVI) is related to cerebral autoregulation (CA), which is the mechanism of the brain to maintain near-constant cerebral blood flow (CBF) despite changes in cerebral perfusion pressure (CPP). Changes in blood vessel impedance enable the stabilization of blood flow. Due to the interplay between CVI and CA, assessment of CVI may enable quantification of CA and may serve as a biomarker for cerebral health.

**Aim:**

We developed a method to quantify CVI based on a combination of diffuse correlation spectroscopy (DCS) and continuous wave (CW) near-infrared spectroscopy (NIRS). Data on healthy human volunteers were used to validate the method.

**Approach:**

A combined high-speed DCS-NIRS system was developed, allowing for simultaneous, noninvasive blood flow, and volume measurements in the same tissue compartment. Blood volume was used as a surrogate measurement for blood pressure and CVI was calculated as the spectral ratio of blood volume and blood flow changes. This technique was validated on six healthy human volunteers undergoing postural changes to elicit CVI changes.

**Results:**

Averaged across the six subjects, a decrease in CVI was found for a head of bed (HOB) tilting of −40  deg. These impedance changes were reversed when returning to the horizontal (0 deg) HOB baseline.

**Conclusions:**

We developed a combined DCS-NIRS system, which measures CBF and volume changes, which we demonstrate can be used to measure CVI. Using CVI as a metric of CA may be beneficial for assessing cerebral health, especially in patients where CPP is altered.

## 1 Introduction

Controlling cerebral perfusion is critical to the health and function of the human brain. Consistency of cerebral blood flow (CBF) is achieved by an adaptive mechanism that occurs in response to changes in cerebral perfusion pressure (CPP).^1^ In this manner, CBF is maintained during physiologic fluctuations, such as those induced by exercise or postural changes.^2^^–^^4^ This adaptive mechanism is termed cerebral autoregulation (CA) and, at the level of relatively slow changes (<0.1  Hz), includes an active vasomotor response to change cerebrovascular impedance (CVI), thus modulating CBF. We propose a novel way of determining CVI as an indirect assessment of CA intactness.

CA has been most often characterized by actively inducing CPP or mean arterial blood pressure (MAP) perturbations and then measuring the CBF response with transcranial doppler ultrasound (TCD),^5^^,^^6^ diffuse correlation spectroscopy (DCS),^7^ or indirectly with near-infrared-spectroscopy (NIRS).^8^ Such perturbations typically include thigh cuff occlusion and rapid release or periodic pressure perturbations, such as paced breathing or sit-stand maneuvers.^2^ CA efficiency is then evaluated as the relative time delay between pressure changes and CBF response.^5^^,^^6^ The active vasomotor response of CA was found only to be active at slow changes (<0.1  Hz) in MAP, with a cut-off frequency of 0.03 Hz.^2^^,^^9^^–^^12^ At higher frequencies, such as cardiac pulsations, no active vasomotor response is observed and changes in CPP are directly translated to changes in CBF. Therefore, the dynamic relationship between the two is driven by vascular compliance and CVI only.^13^^,^^14^

CVI is a frequency-dependent variable, which is calculated as the spectral ratio of CPP to CBF. In comparison, cerebrovascular resistance (CVR) is the steady state frequency-independent equivalent, which assumes there are no temporal changes between pressure and flow. Like CVR, CVI relates vascular pressure dynamics to vascular flow dynamics.^13^^,^^14^ However, CVI can account for parameters that would otherwise invalidate CVR calculations, such as vessel compliance, blood inertia, or frequency-dependent changes in either parameter.^13^^,^^15^

Classically, CA, and thus CVI, assessment relies on quantifying vasomotor dynamics at slow frequencies.^16^ Here, we propose to utilize strong hemodynamic changes naturally generated in the body at a semi-constant rate—namely, the heartbeat, which does not have an active vasomotor response and relies only on vessel compliance. Although CA is not directly acting on high frequencies such as cardiac pulsation, CVI is a function of baseline CPP, as described by Lassen’s curve^2^^,^^17^^,^^18^ and thus an indirect marker of CA intactness. By focusing on cardiac pulsatile blood flow and blood pressure waveforms naturally occurring in the body, we also circumvent the need to induce MAP or CPP changes to estimate CA; therefore, no active participation of the subjects is required.

When evaluated at the cardiac pulsation, CVI requires high temporal resolution measurements of pulsatile blood flow and blood pressure changes. TCD combined with pulsatile blood pressure from the finger^15^ or femoral artery^13^^,^^14^^,^^19^ has successfully determined CVI, but lacks the capacity for continuous monitoring over long periods. Here, we present a method to estimate CVI changes as the first step toward an impedance-based CA assessment. For this, we use diffuse optical imaging, which can be used continuously at the bedside. We will report on a new colocalized high-speed DCS-NIRS design that can record high temporal resolution blood flow and blood volume changes in the microvasculature directly on the head. Afterward, we evaluate here healthy human volunteers undergoing full body tilts to induce CVI changes and present a novel method to calculate these CA-driven CVI changes.

Because NIRS and DCS are methods that can be used at the clinical bedside and continuously,^20^^,^^21^ we believe that the presented approach will be beneficial in disease monitoring where CA is known to be impaired, including stroke,^22^^–^^25^ traumatic brain injury,^22^^,^^26^ and hydrocephalus.

## 2 Methods

To develop a method to record CVI, we first designed a new, combined, and high-speed DCS-NIRS system. We then ran two separate studies. Experiment 1 validates the relationship between pulsatile blood volume and pulsatile blood pressure. Experiment 2 is a separate experiment to measure CVI during head of bed (HOB) tilting.

### 2.1 Design of a Combined DCS-NIRS System

To calculate impedance using pulsatile hemodynamics, we developed a combined DCS-NIRS system, which operated at 500 Hz sampling rate and measures colocalized blood volume and blood flow changes. The source-detector distance was set to 2 cm. The DCS device is based on a software correlator approach and uses a long coherence length fiber-coupled laser operating at 785 nm (DL785-70-3O, Crystalaser, Reno, Nevada, United States). The output power was reduced to 30 mW using a manual fiber attenuator (VOAMMF, Thorlabs, Newton, New Jersey, United States). The light was coupled to a 400-μm multimode (MM) fiber for the illumination of tissue. Collection of back-scattered light was done using a bundle of four “few-mode” (FM) fibers (980HP, Thorlabs) to increase SNR^27^ and detected with a four-channel single photon avalanche detector (SPAD) module (SPCM-AQ4C, Excelitas, Waltham, Massachusetts, United States). The digital pulses from each detector are received by an field programmable gate array (FPGA; XC7A100T-2CSG324C, Xilinx, San Jose, California, United States). The FPGA is internally configured as an asynchronous counter that increments an internal 3-bit counter, per channel. The value of the asynchronous counter is recorded at 10 MHz, yielding a bin size of 100 ns.

**Fig. 1:**
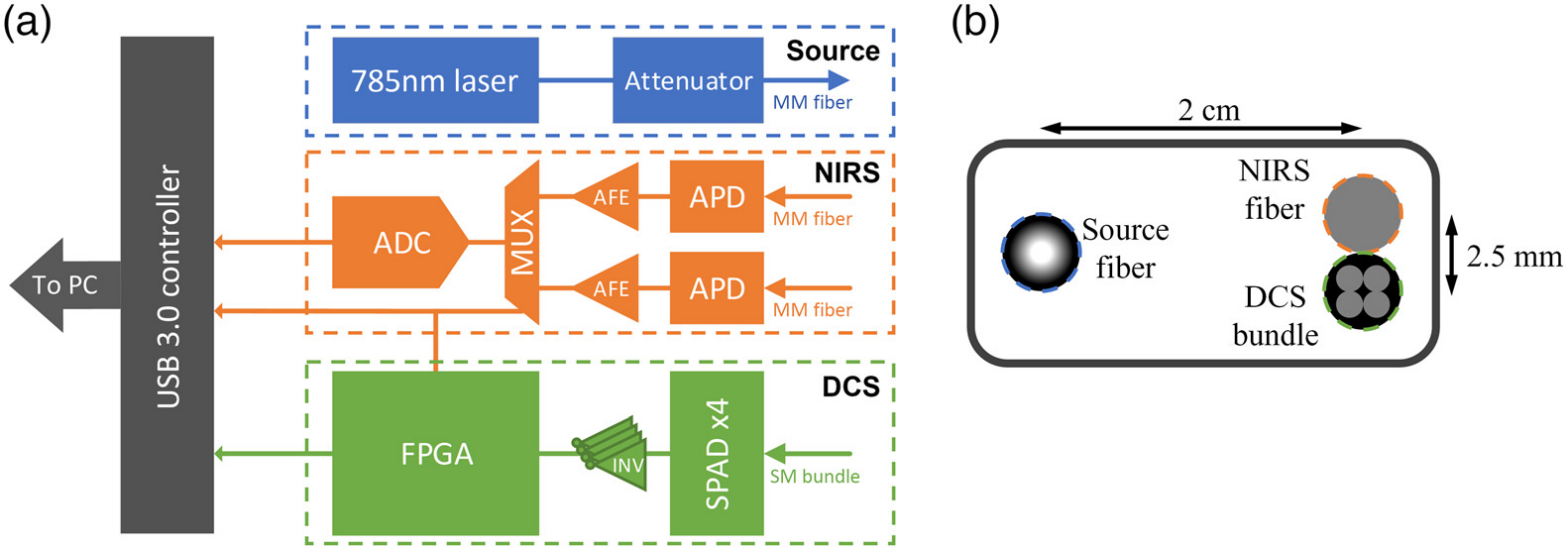
(a) High-level system block diagram of the combined DCS-NIRS system. A laser source with an attenuator coupled to an MM fiber is shown in blue. NIRS signals are received through an MM fiber, filtered using a custom AFE then time-interleaved using an MUX, and sampled using a single ADC shown in orange. DCS signals are received through an FM fiber bundle and sent to the SPAD array. The SPAD detectors emit an electrical pulse that is then buffered and counted using an FPGA shown in green. Data are placed on a bus to be sent to the computer shown in gray. (b) Probe design schematic showing relative fiber positions and spacing.

The NIRS detection was colocalized with the DCS, using the same laser for illumination. A 200-μm MM fiber was placed tangential to the DCS detector fibers where the 2.5-mm ceramic ferrules were touching. The distance between the centers of the DCS fiber bundle and NIRS detector fibers was 2.5 mm and thus probes the same location as DCS for diffuse applications. The light was detected using a temperature-compensated avalanche photodetector (APD410A, Thorlabs). A custom analog front-end (AFE) amplified and low-pass filtered the signal with a cut-off frequency of 100 Hz. Two detectors and two AFEs were used to allow for averaging of multiple detectors together, or for simultaneous measurements at two different detector locations depending on the experiment. These signals were time-interleaved using an analog multiplexer (MUX), then digitized using a 14-bit analog-digital converter (ADC) operating at the same 10-MHz FPGA sampling clock. A high-level schematic of the DCS NIRS device and probe design is shown in Fig. 1. Two identical optical probes were built for simultaneous acquisition of the periphery and the head.

DCS and NIRS data were collected and streamed to a computer using a USB 3.0 controller configured as a high-speed acquisition system. The raw DCS and NIRS data were recorded at 10 MHz for postprocessing. The instrument also contained a custom electrocardiogram (ECG) device that recorded the onset of the R-peak of the ECG waveform. The R-peaks are the highest amplitude component of the ECG waveform and signify the onset of ventricular depolarization.^28^ The timing information provided by the ECG R-peaks allowed for averaging several (∼100) blood flow and volume pulses to increase the fidelity of the cardiac waveform.

### 2.2 Human Subject Experiments

Flow impedance is the frequency-dependent analog of flow resistance and can be calculated as

$$Z(j\omega )=\frac{P(j\omega )}{Q(j\omega )},$$ (1)

where P(jω) is the frequency-dependent fluid pressure drop across a vessel and Q(jω) is the frequency-dependent flow through the vessel.^13^^,^^14^ In the periphery, P(jω) is pulsatile arterial blood pressure (ABP). In the cerebral environment, P(jω) is pulsatile CPP defined as the difference between pulsatile ABP and intracranial pressure (ICP).

Although we could measure pulsatile blood flow using our DCS device, we did not have a way to directly measure pulsatile blood pressure changes in the same location. To address this, we first ran a study to investigate the relationship between pulsatile pressure changes and pulsatile blood volume changes measured with NIRS in the arm. We recruited four healthy human volunteers and measured a linear relationship between changes in blood volume and pressure at the cardiac pulsation due to the constant elastic modulus of vessels over small distentions.^29^

After validating this relationship, we used NIRS-based pulsatile volume changes as a surrogate for pulsatile pressure changes in a second experiment to measure cerebral impedance. We measured impedance at the cardiac frequencies and harmonics on six healthy volunteers.

#### 2.2.1 Experiment 1: pulsatile blood pressure versus volume in the arm

**Fig. 2:**
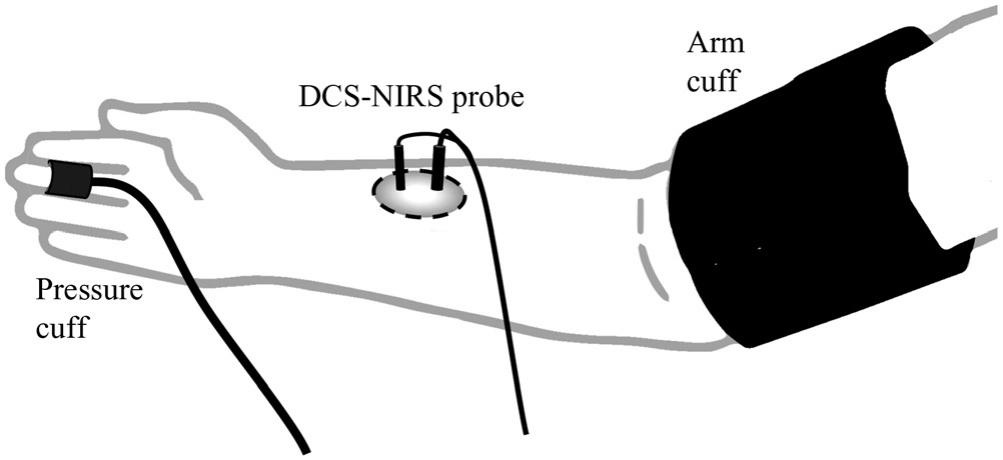
Experimental setup and probe placement for validation of pulsatile pressure-volume relationship. A pressure cuff was placed on the arm to modulate pulsatile pressure through arm occlusions. A DCS-NIRS probe and a noninvasive pressure monitor were placed on the arm and finger, respectively to record hemodynamics. ECG leads (not shown) were placed on the chest to provide a timing signal for postprocessing.

The experimental protocol was approved by the Carnegie Mellon University Institutional Review Board (IRB #2015 00000113). Written informed consent was obtained from all participants before the study. The DCS-NIRS probe was placed on the forearm above the brachioradialis muscle, as seen in Fig. 2. Only the NIRS system was activated to measure pulsatile blood volume changes. A continuous noninvasive blood pressure monitor (CNAP 500, CNSystems, Graz, Austria) was placed on the finger of the same arm to record pulsatile blood pressure. Our ECG monitor was placed on the chest to provide the timing of the cardiac cycle. A separate blood pressure cuff was placed on the same arm to modulate the pulsatility in blood pressure through partial arm occlusions. The pressure cuff was inflated such that the pulsatility (difference between systolic and diastolic blood pressure) reported by the CNAP blood pressure monitor was approximately 10 mmHg for 2 min, followed by 20 and 30 mmHg. Baseline measurements of 5 min at the subjects’ nominal MAP were taken before and in between cuff inflation.

#### 2.2.2 Experiment 2: impedance changes in head and arm

**Fig. 3:**
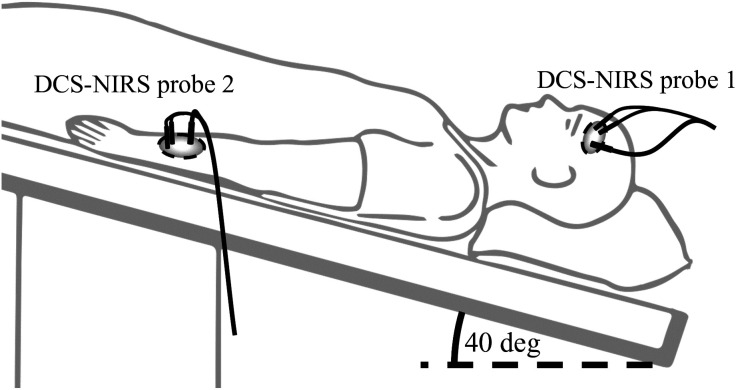
Experimental setup for impedance measurements. Subjects’ entire bodies were tilted head down to induce impedance changes in the head. A DCS-NIRS probe was placed on the forehead, 2 cm above the left eyebrow, and the arm above the brachioradialis.

Under the same IRB protocol, an additional six healthy volunteers were enrolled in a HOB tilting study. Gravity and posture changes can perturb CA through baseline changes in CPP, thereby causing a change in impedance in the head.^30^^–^^34^ Using an inverting table, subjects initially lay flat at 0 deg for 15 min, followed by a full body tilt downwards to −40  deg for 15 min, then returning to 0 deg for another 15 min. A DCS-NIRS probe was placed on the forehead, 2 cm above the left eyebrow, to measure pulsatile blood flow and volume changes. An additional, identical DCS-NIRS probe using a second 785-nm laser was placed on the arm, above the brachioradialis muscle. The arm location served as a control measurement because CA does not exist in the periphery. Thus no impedance changes were expected in the arm. The experimental setup is shown in Fig. 3.

### 2.3 Data Analysis

After the DCS-NIRS data were streamed to a computer, data were processed using a standard Linux-based computer running the Python programming language (python.org). The custom Python program saves the unprocessed binary data from the device directly to the disk while performing temporal autocorrelations and curve fitting and displaying the resulting DCS flow and NIRS data at >50  Hz.

#### 2.3.1 DCS and NIRS data analysis

The fluctuations of the electric field of correlated speckles contain dynamic information about the moving scatterers.^35^^–^^38^ This dynamic information at a particular time t can be empirically extracted using the electric field temporal autocorrelation function

$$G_{1}(\tau )=\langle E(t)\cdot E^{*}(t+\tau )\rangle ,$$ (2)

where ⟨…⟩ denotes a temporal average and E and   τ represents electric field amplitude and delay time, respectively.^37^ $G_{1}(\tau )$ can be solved analytically using the correlation diffusion equation, which holds the flow-dependent variable, $\alpha D_{B}$. For practical implementations of DCS, intensity autocorrelations (rather than electric field autocorrelations) are measured. The electric field autocorrelation can be derived from the normalized intensity autocorrelation ($g_{2}$) through the Siegert relation

$$g_{2}(\tau )=1+\beta \cdot \frac{{\left|G_{1}(\tau )\right|}^{2}}{{\left\langle I(\tau )\right\rangle }^{2}},$$ (3)

where ⟨I(τ)⟩ is the time average intensity and β is an autocorrelation contrast parameter that depends on the experimental setup, particularly the number of independent speckles detected and the coherence length of the laser used.^27^^,^^36^^,^^37^^,^^39^^,^^40^

Intensity autocorrelations $g_{2}(\tau )$ for each channel of the DCS data were calculated using an integration window of 20 ms and a window overlap of 18 ms. The SNR for every delay time was calculated over the entire measurement using the equation

$$\mathrm{SNR}(\tau )=\frac{\overset{¯}{g_{2}(\tau )}-1}{\sigma_{g_{2}}(\tau )},$$ (4)

where $\bar{g_{2}(\tau )}$ is the mean temporal autocorrelation per delay time and $\sigma_{g_{2}}(\tau )$ is the standard deviation per delay time over the measurement duration.^38^^,^^41^

To extract blood flow, we fit the correlation diffusion equation to each $g_{2}$ curve assuming nominal values of $\mu_{a}$ and $\mu_{s}$ of 0.1 and $10\;{\mathrm{cm}}^{-1}$, respectively.^42^ Fitting was done for β and the flow-dependent variable, $\alpha D_{b}$, simultaneously. $\alpha D_{b}$ was converted to a blood flow index (BFI) by normalizing $\alpha D_{b}$ to the mean flow, then scaling it to a percentage. A custom Python function was used to perform a nonlinear least-squares fitting using the SNR as a weighting function for each delay time. This weighting function was implemented to reduce the effect of $g_{2}$ measurement noise on the fitted curve. The choice for the $g_{2}(\tau )$ integration window and overlap resulted in a blood flow sample rate of 500 Hz and a signal bandwidth of 50 Hz. 50 Hz was selected as it provides sufficient temporal bandwidth to capture the entire pulsatile flow waveform including all of the higher-order harmonics for even the fastest heart rates. By having a sample rate of 500 Hz, sample binning and averaging can be implemented to further increase the signal SNR.

For NIRS, the intensity data were averaged and downsampled to match the output sample rate and bandwidth of the DCS data; 500 and 50 Hz, respectively. The modified Beer-Lambert law was applied for calculating changes in absorption coefficient ($\Delta \mu_{a}$).^43^ Because our device was operating near the isosbestic point of hemoglobin,^44^^–^^46^ we assumed that absorption coefficient changes are proportional to total hemoglobin concentration changes ΔHbT. Total hemoglobin changes were used to indicate blood volume changes.

#### 2.3.2 Microvascular blood pressure to blood volume relationship analysis (experiment 1)

To evaluate the linearity between pulsatile ΔHbT and pulsatile blood pressure, the data was first bandpass filtered between 0.35 and 35 Hz using cascaded high and low-pass eighth-order Butterworth filters to remove respiration and high-frequency instrument noise, respectively. The lower cutoff frequency and filter order were selected to offer a sharp transition between the respiration and heart rate frequencies. The upper cutoff frequency was selected to recover all harmonics of the cardiac pulsation while rejecting environmental electrical interference, such as the 60-Hz powerline frequency.

To avoid any effects from nonstationary hemodynamics caused by blood pooling immediately after a partial arm occlusion, only the last 30 s of each trial (baseline and each partial occlusion) were taken for further analysis. This provided sufficient time to ensure that the arm reached a hemodynamic steady state. Each pulse in the ΔHbT and blood pressure data was aligned to the R-peaks generated by the ECG and averaged to generate an average pulse waveform. The magnitude of this averaged pulse waveform was determined by peak-to-peak amplitude. We rejected any trial where the standard deviation of all the pulses exceeded the magnitude of the averaged pulse. To calculate percent changes in HbT, we assumed a baseline value of 60  μM of hemoglobin.^47^

#### 2.3.3 CVI calculation during HOB tilting (experiment 2)

**Fig. 4:**
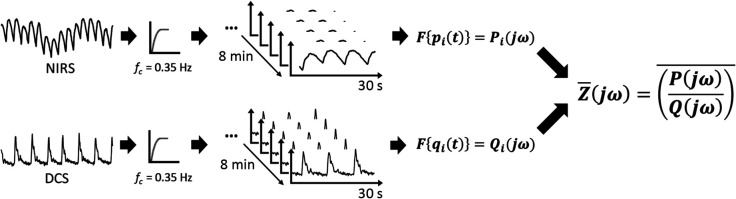
Overview of signal processing from DCS-NIRS data to impedance per angle. DCS and NIRS signals were high-pass filtered to remove unwanted signals and then separated into overlapping 30-s windows. The Fourier transform was taken for each window, and all windows were averaged together to determine the spectral flow and pressure, respectively. Impedance was calculated by the ratio of pressure and flow.

The data processing approach for impedance extraction is shown in Fig. 4. We restricted our analysis only to the cardiac frequencies such that we could assume the subjects’ autoregulation system is stable. We applied an eighth-order Butterworth filter with a cutoff frequency of 0.35 Hz to remove the influence of respiration and spontaneous fluctuations in both BFI and HbT measurements. The last 8 min of data per tilting angle were used for further processing to reduce the initial transient effects of posture changes. Data were segmented into 30-s windows with a 29-s overlap. Based on the results of experiment 1, we used pulsatile HbT as a surrogate measurement for pulsatile pressure changes. The Fourier transform of each time window was applied to obtain the spectral power of pressure (using HbT) and BFI. Impedance was calculated by applying Eq. (1) to each time window.

We calculated the impedance spectra at the cardiac pulsation frequency and its harmonics, and we linearly interpolated the spectral data over the frequencies where there is no cardiac spectral energy. To determine the spectral regions to interpolate, we defined an SNR metric

$${\mathrm{SNR}}_{Z}(\omega )=\frac{\overset{¯}{\left|Z(j\omega )\right|}}{\sigma (|Z(j\omega )|)},$$ (5)

where $\bar{\left|Z(j\omega )\right|}$ is the average magnitude of impedance, averaged over all time windows, and σ(|Z(jω)|) is the standard deviation of the magnitude over all time windows. We replaced frequencies of ${\mathrm{SNR}}_{Z}(\omega )<1$ with interpolated spectral content between cardiac harmonics.

Because we did not have access to absolute CPP, we calculated relative changes in impedance, rather than absolute impedance curves. To allow for inter-subject comparisons, subject-dependent scaling factors, such as the pulsatile pressure-volume coefficient, must be normalized out from each subject’s set of impedance curves. This was done by dividing all the curves from a particular subject by the subject’s impedance at the heart rate frequency for the initial 0 deg angle. The location of this fundamental heart rate frequency was determined by taking the location of the average peak of the blood flow spectra across the 30 s windows at the 0 deg angle.

Impedance was also visualized in the time domain as a conductance impulse response defined as

q(t)=p(t)*g(t), (6)

where q(t), p(t), and g(t) are temporal flow, pressure, and the conductance convolutional kernel (impulse response), respectively. This convolutional kernel can be derived from impedance as

$$g(t)=F^{-1}\{\frac{1}{Z(j\omega )}\}.$$ (7)

Taking the average impedance spectra without interpolation, we applied Eq. (7) to estimate g(t). We then applied an eighth-order Butterworth low-pass filter with a cutoff frequency of 35 Hz to remove high-frequency noise artifacts caused by our signal processing from our estimate. We used the spectra found prior to interpolation to avoid discontinuities in the spectra which would cause artifacts when taking the inverse Fourier transform.

## 3 Results

### 3.1 Relationship Between Pulsatile Blood Pressure and Volume Changes (Experiment 1)

**Fig. 5:**
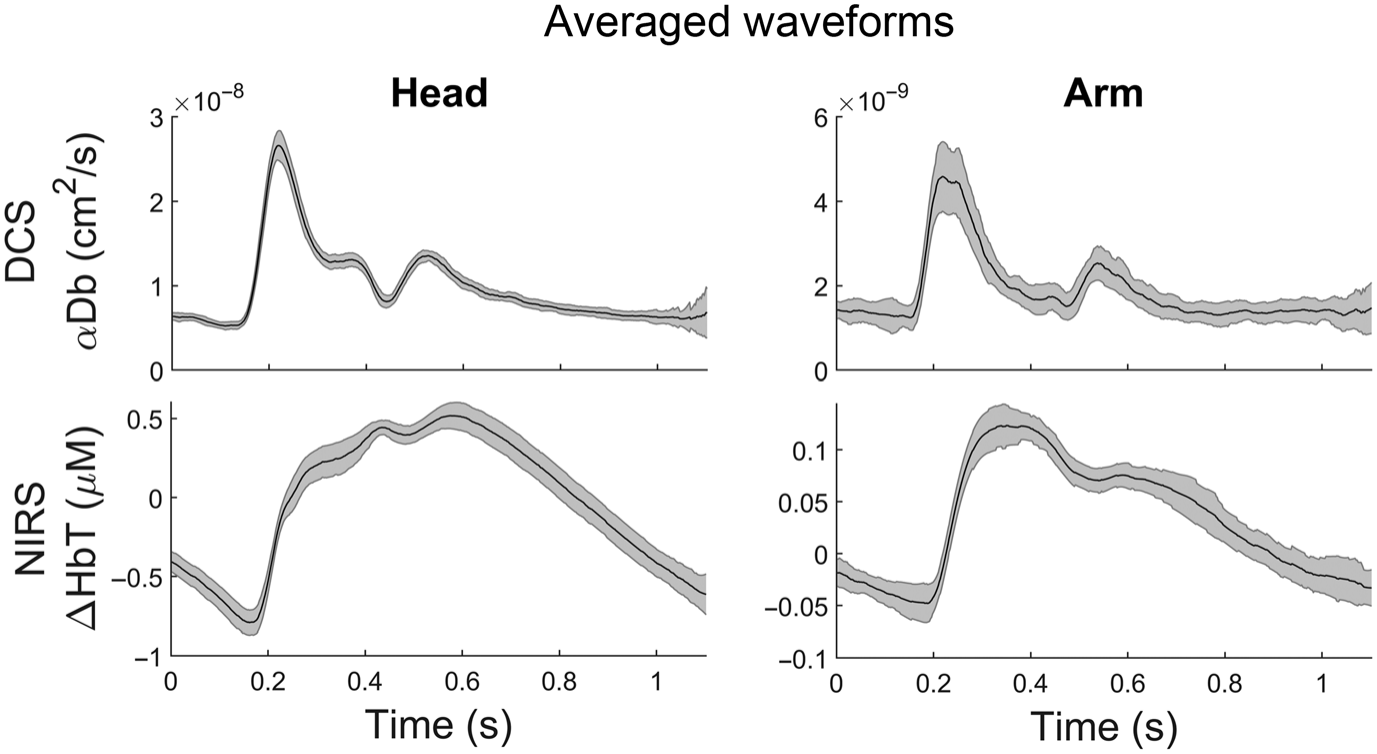
Sample DCS-NIRS curves for head and arm. Data show the resulting average of ∼100 individual cardiac pulsations sampled at 500 Hz. The shaded area represents the standard deviation from the mean.

An example of averaged pulsatile blood flow and blood volume data is seen in Fig. 5 showing DCS and NIRS measurements in the head and arm averaged over approximately 100 cardiac cycles. Data were aligned to the ECG R-peak and averaged. The high sampling rate of our DCS-NIRS device provides sufficient temporal resolution to resolve the dicrotic notch in the ECG-averaged cerebral waveforms, as seen in Fig 5.

**Fig. 6:**
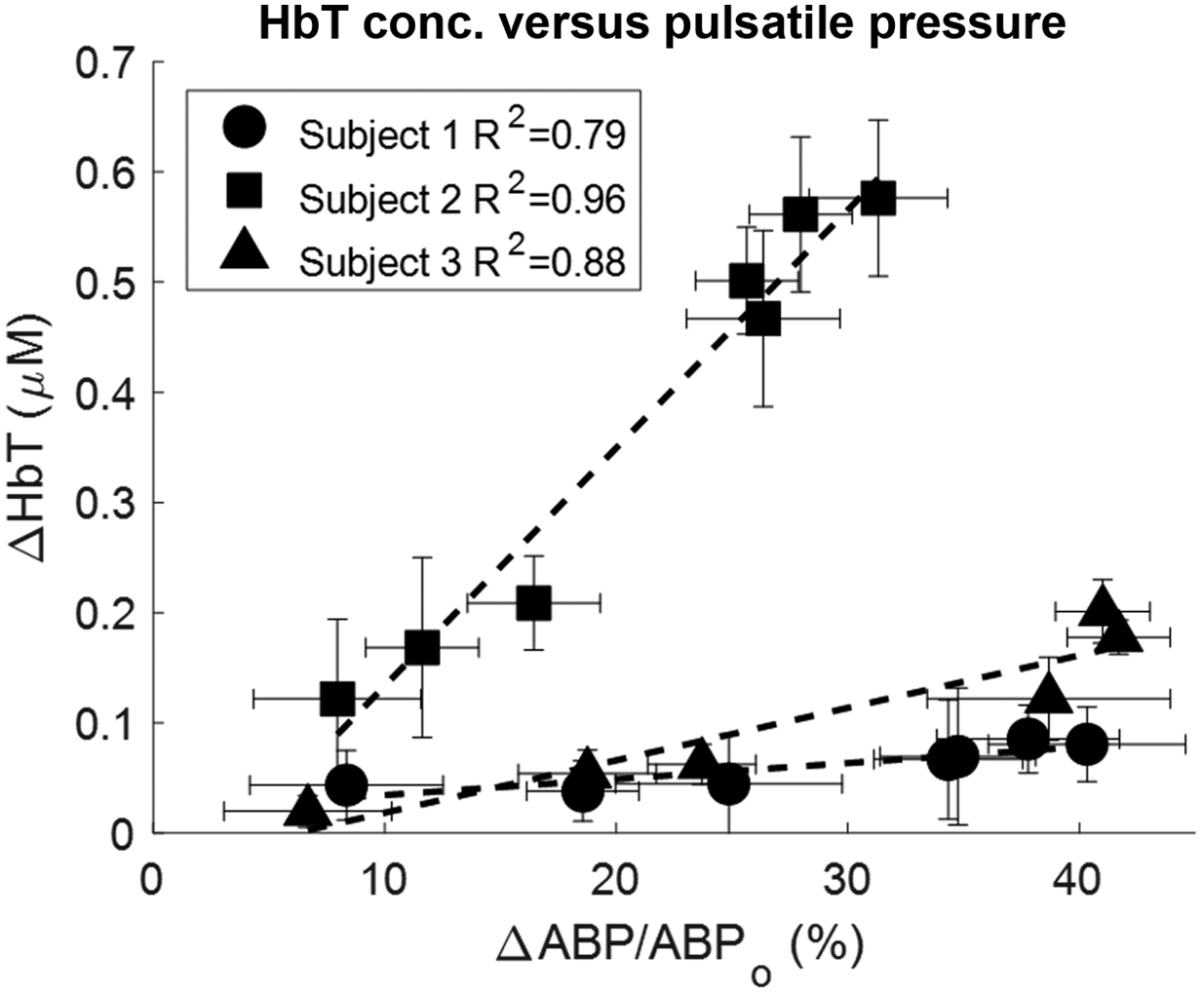
Scatterplot of pulsatile volume changes as a function of pulsatile pressure changes. A linear fit was performed for each subject and $R^{2}$ was determined. Error bars denote the standard deviation from the mean.

Because impedance is defined as the spectral ratio between blood pressure across the vessel and blood flow, we estimated pressure changes by assuming a linear relationship to blood volume changes. To show this, we quantified the magnitude of the pulsatile blood pressure and the magnitude of pulsatile blood volume in the periphery. The relationship between the two is seen in Fig. 6, which shows a scatter plot of the magnitude of blood volume changes as a function of blood pressure changes. The results for three subjects are shown; data from the fourth subject was rejected based on our SNR criteria. At the baseline blood pressure, all subjects showed a pulsatility of 30 to 40 mmHg. A linear relationship was fitted between pulsatile blood pressure and blood volume for the three subjects resulting in $R^{2}$ values of 0.79, 0.96, and 0.88 for each subject. Because our system cannot resolve absolute hemoglobin values, changes in pulse magnitude are relative and slopes between subjects are subject-specific. Based on the linearity found in Fig. 6, we used ΔHbT(t) as a surrogate for ΔP(t) for further analysis.

### 3.2 Cerebrovascular Impedance and Impulse Response During HBO Tilting (Experiment 2)

**Fig. 7:**
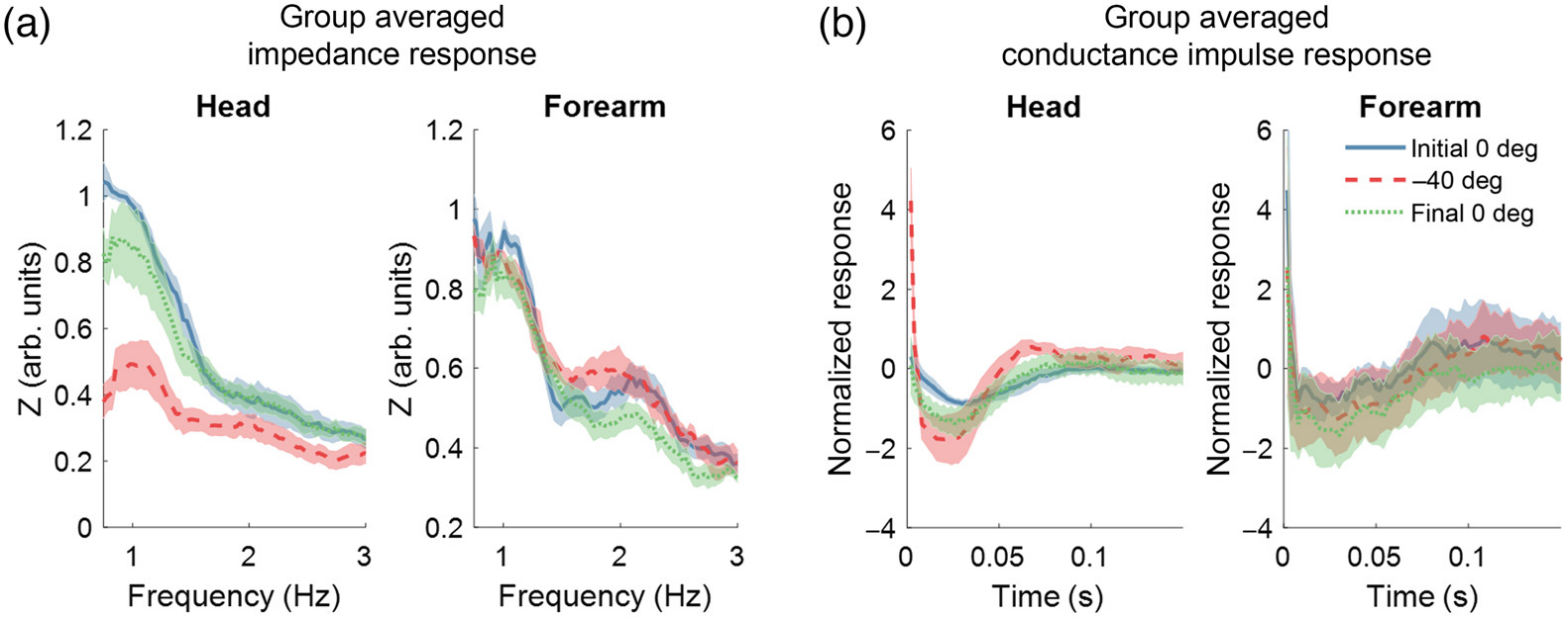
(a) Normalized group averaged (N=6) impedance spectra and (b) conductance impulse response for the head and arm DCS-NIRS locations. Each color represents a different HOB angle. Shaded areas signify standard error from the mean.

Using the linear relationship between blood pressure and blood volume at the cardiac pulsation found in experiment 1, we calculated CVI for each tilt angle based on Eq. (1), using Q=BFI and P=ΔHbT. Figure 7(a) shows the group average impedance curves across all subjects (N=6), with error bars representing the standard error from the mean. There was a negligible change in the heart rate during the experiment, with the average heart rate across all six subjects at the baseline being 61.35 BPM, which increased to 61.49 BPM during the −40  deg tilt and decreased to 61.44 BPM during the recovery. Average cerebral BFI increased by 140% at −40  deg relative to the initial baseline, then decreased to 108% when returning to 0 deg. The results show that the average impedance across subjects between 1 and 2 Hz is lower in the head at −40  deg when compared to the 0 deg angles. In contrast, the arm impedance spectra show minor changes over HOB tilting angles [Fig. 7(a)].

Figure 7(b) shows the normalized group averaged conductance impulse response for each detector location. Like the trends in the impedance spectra, the data show an increase in impulse amplitude in the response curve of the head when tilted downwards to −40  deg when compared with the 0 deg angles. Likewise, there is little morphological change in the impulse response in the arm when tilting.

## 4 Discussion

### 4.1 Design of a High-Speed DCS-NIRS System

Our combined high-speed DCS-NIRS system can simultaneously measure pulsatile, colocalized blood volume, and blood flow. Using ECG-timed pulse averaging, we could further increase the fidelity of hemodynamic pulsatile signals. Such high-resolution signals can subsequently allow for deeper insight into tissue’s physiological status. Several prior studies have investigated the combination of DCS and NIRS to measure cerebral hemodynamics.^48^^–^^53^ However, many of these studies employed the use of hardware correlation and/or frequency domain NIRS, which restrict sample rates to ∼1 to 10 Hz, which may not provide sufficient temporal bandwidth to reconstruct the higher-order harmonics of the cardiac signals and thus cannot resolve the finer details of the cardiac waveforms. Our device, in combination with ECG alignment, can provide sufficient temporal resolution and SNR to analyze several harmonics of the pulsatile waveform. Furthermore, many DCS-NIRS implementations rely on separate DCS and NIRS devices, which present alignment and optical crosstalk challenges which limit temporal resolution.^48^ By combining DCS-NIRS hardware into a unified package and sharing a common laser source, our device circumvents alignment and optical crosstalk challenges, which allow reliable reconstruction of pulsatile hemodynamics.

### 4.2 Local Blood Pressure Estimation

For this work, we have assumed a linear relationship between pulsatile blood volume and pulsatile blood pressure changes based on data collected in the arm, with data shown in Fig. 6. This indicates a constant elastic modulus for the blood vessel bed during a cardiac pulsation.^29^ Pulsations in the NIRS signal are due to volume expansions in the vessels from pulsatile pressure changes. In the arm, these pulsatile volume changes are due to pulsations in ABP. However, in the head, the effects of ICP must be considered. Pulsatility in ICP is a passive response to pulsations in ABP.^54^^,^^55^ These pulsations in ICP can reduce the magnitude of the CPP pulsations, which drive vessel dilation in the brain. Due to the passive nature of pulsations in ICP, we assumed that NIRS-reported pulsatile blood volume change is linear to CPP. Therefore, we assumed that the linearity between blood volume and CPP pulsations still holds for cerebral measurements. Although the pulsatile pressure-volume slope in the brain may differ from the arm, the normalization method in CVI calculation removes the subject-dependent slope and is therefore still valid.

Exponential relationships in vessels can occur at extreme pressure and volume changes where vessel elastic-modulus is no longer constant.^29^ To ensure that our impedance trends were still consistent using a nonlinear pulsatile pressure-volume relationship, we ran our analysis assuming a power law relationship ($\Delta P=k^{\Delta \mathrm{HbT}}$ where k=10). We found (data not shown) that the impedance curve trends with tilting angle were qualitatively the same as when making the linearity assumption. This shows that our method is not confounded by a nonlinear pressure-volume assumption.

### 4.3 Agreement of Impedance and Autoregulation in Posture Changes

Due to the high sampling frequency for our NIRS and DCS device, harmonics of the pulsatile hemodynamics could be used for the estimation of vascular impedance. Posture changes have been shown to affect CPP, which trigger an autoregulative response^3^^,^^56^ and subsequently induce impedance changes. Literature shows that CPP decreases in Trendelenburg positioning (head down tilt).^32^^,^^33^^,^^57^ At a −40  deg tilt, we expected a decrease in CPP, which would cause vasodilation in the cerebral microvasculature, thereby causing a decrease in CVI when compared to 0 deg. In contrast, we expect that HOB tilting would not affect the CVI in the periphery. Our results shown in Fig. 7(a) resemble previously reported trends in larger vessels where vasoconstriction leads to an overall increase in impedance and, conversely, vasodilation results in a lowering of impedance.^3^^,^^13^ Thus, it corroborates the fact that we observe a decrease in impedance when tilted downwards as vessels must dilate to maintain sufficient perfusion in the face of decreasing CPP.

### 4.4 Limitations and Future Work

We have shown that changes in impedance can be recorded as a function of the HOB tilting angle. However, this method is unable to quantify absolute impedance due to the subject-specific pressure-volume proportionality coefficient, which limits the estimation of absolute pulsatile pressures. This may be caused, in part, using continuous-wave NIRS, which is only able to measure blood volume changes rather than absolute values. Despite our impedance estimation being a relative measurement, we have found that normalizing the impedance curve using the heart rate frequency allows for inter-subject comparisons.

For this work, we assumed our HOB tilts induced changes in CPP, which triggered a CA response. However, our study is not able to directly quantify these CPP changes without the use of invasive pressure probes. Future controlled invasive experiments using ICP and ABP pressure probes are needed to measure CPP and quantify the relationship between CPP and CVI. Furthermore, our approach is only applicable when we can assume that impedance is quasi-constant throughout each tilt angle measurement. This notably has implications for the impedance frequencies we can estimate. In the brain, low-frequency changes are modulated by CA.^9^^–^^12^ Thus, the impedance at these frequencies will vary over time and cannot be estimated using our method. However, our approach assumes that the cardiac frequencies are not actively regulated through active vasodilation or constriction to counteract pressure changes. We therefore can only estimate vascular impedance at higher frequencies such as the cardiac pulsation.

Finally, our device is operating at a single wavelength close to the isosbestic point, namely at 785 nm.^44^ Whereas this operation allows for an estimation of total hemoglobin changes, we were not able to differentiate between oxyhemoglobin and deoxyhemoglobin. A future iteration of this device will incorporate multiple wavelengths to extract information about different hemoglobin species. To achieve this, a multicore source fiber could be used for illumination where each core contains a separate wavelength. At the detector end, the DCS fibers and two NIRS fibers could be bundled together to enable colocalization of the NIRS and DCS signals across the different wavelengths and optical filters used for wavelength selectivity.

## 5 Conclusion

Here, we have found a linear relationship between changes in blood volume and blood pressure at the cardiac pulsations, allowing us to use blood volume changes as a surrogate for blood pressure changes at that frequency. Additionally, we have demonstrated a method to estimate changes in CVI from postural changes in healthy subjects using our high-speed DCS-NIRS system. These changes in CVI and the mathematically similar conductance impulse response were consistent with the expected CPP change and resulting autoregulative response. To achieve this, we have demonstrated a design for a DCS-NIRS system that enables high temporal resolution hemodynamic recordings and is immune to synchronization and crosstalk limitations. Overall, this device and technique may enable further insight into the mechanics of CA and provide a path forward to quantifying autoregulative function.
